# Secondary Vasculitis Attributable to Post-COVID Syndrome

**DOI:** 10.7759/cureus.44119

**Published:** 2023-08-25

**Authors:** Kelly M Frasier, Caroline Gallagher-Poehls, Mikayla Cochrane, Debosree Roy

**Affiliations:** 1 Public Health, AT Still University - School of Osteopathic Medicine, Mesa, USA; 2 Public Health, Sidney Kimmel Medical College at Thomas Jefferson University, Philadelphia, USA; 3 Research, A.T. Still University School of Osteopathic Medicine, Mesa, USA

**Keywords:** coronavirus, temporal arteritis, vasculitis, type iii hypersensitivity, covid-19, vasculopathy, thrombosis, sars-cov-2, hemodynamics, endothelium

## Abstract

While the acute phase of the COVID-19 pandemic has largely come to pass, the chronic physiologic effects of the coronavirus continue to unfold. Specifically, the number of COVID-19-associated vasculitis cases has steadily increased since the onset of the pandemic. Data have shown that vasculitis may develop less than two weeks after COVID-19 or during a later onset of the disease. At this time, research has demonstrated that the novel coronavirus invades more than just the lungs; it can also attack the nervous system, cardiovascular system, and kidneys. In addition, there is a greater understanding of the pathogenesis regarding COVID-19-induced vasculitis via humoral immunity and immune complex disease. Recent case reports have shown an association between COVID-19 and secondary vasculitis. This review paper discusses case reports and data that suggest that COVID-19 may lead to specific vasculitis diseases such as giant cell arteritis, ophthalmic arteritis, aortitis, and Kawasaki-like disease. More research needs to be performed on this association to aid in diagnosis and treatment.

## Introduction and background

The coronavirus disease 2019 (COVID-19) pandemic has now totaled 102,977,396 cases and more than 1,120,529 deaths in the United States as of April 2023 [1]. The severe acute respiratory syndrome coronavirus 2 (SARS-CoV-2) virus has proven to have high transmissibility and host infectivity with the ability to affect the circulatory system. It has become evident that SARS-CoV-2 affects the circulatory system via tissue tropism or secondary inflammatory responses via innate immunity. Furthermore, indirect inflammatory responses to SARS-CoV-2 result from leukocyte debris like cell-free deoxyribonucleic acid (DNA), histones, and ribonucleic acid (RNA) viral particles [2]. A case series of 65 articles reported that the most common post-COVID-19 complications are lung injuries, venous or arterial thrombosis, heart injuries, cardiac or brain strokes, and neurological injuries; however, vasculitis associated with COVID-19 has both acute and chronic health implications [3]. More research is needed in this area to determine the degree to which inflammation caused by SARS-CoV-2 attributes to various types of both large- and small-vessel vasculitis. Overall, the most common vasculitis associated with COVID-19 patients is leucocytoclastic (LCV), immunoglobulin A (IgA), and Kawasaki disease [4].

## Review

Vasculitis is commonly considered an organ-specific immune-related complication of a COVID-19 infection seen in post-COVID syndrome. The number of COVID-19-associated vasculitis cases is gradually increasing [5]. As research has found specific inflammatory immune responses triggered by the COVID-19 infection, both acute and chronic forms of vasculitis are slowly becoming more heavily evaluated. It has been found that vasculitis may occur early following the onset of COVID-19 (an interval of <two weeks) or develop later during or after the course of the disease with an associated significant morbidity [5]. Since COVID-19-associated vasculitis has the potential to cause substantial outcomes in both morbidity and mortality, it remains imperative to further explore this organ system and how it correlates to SARS-CoV-2 and create a better understanding of how to recognize and treat this specific subtype of secondary vasculitis.

So far, preliminary research has found that many patients previously diagnosed with COVID-19 experience additional clinical manifestations and cases of isolated vasculopathy [6]. The association between SARS-CoV-2 direct or indirect vasculopathy and a correlation with disease prognosis has not yet been fully discovered. At this time, evidence and data suggest that the novel coronavirus, aside from its primary respiratory involvement, can also invade vascular endothelial cells of several systems throughout the body, including cerebral, cardio-pulmonary, renal microvasculature, and multiple visceral perfusion indices [6]. Additionally, it has recently been proven that COVID-19 directly causes vasculitis via a specific immune response. Notably, research has provided scientific evidence that in COVID-19 vasculitis, there appears to be a life-threatening escalation from type 2 T-helper immune response (humoral immunity) to type 3 hypersensitivity (immune complex disease) [7]. Subsequently, the deposition of immune complexes was seen inside vascular walls that induced a severe inflammatory state [7]. The immune complexes within the vasculature trigger an immune response, leading to varying types of vasculitis throughout the body. Roncati, L. et al. found a cytokine release syndrome, where interleukin-6 (IL-6) is the main myokine, in the smooth muscle cells found in blood vessels [7].

Recent evidence has suggested cutaneous vasculitis (CV) may occur during or after the COVID-19 infection. Cutaneous vasculitis is a skin-limited, inflammatory disease of the vasculature that affects both dermal and hypodermal vessel walls. Vasculitis affecting the skin may result from drugs, vaccines, and infections. Cutaneous vasculitides are defined as a group of inflammatory disorders affecting blood vessels within the skin [8]. Cutaneous small-vessel vasculitis has been the most commonly reported dermatological vasculitis in COVID-19 patients [9]. Cutaneous vasculitis, previously known as leukocytoclastic vasculitis, occurs both with COVID-19 infection, vaccination, and post-COVID syndrome [9]. The mechanism of action seen in vasculopathy on the histology of patients with severe infection appears to be endothelial injury due to immune thromboembolic mechanisms. The pathogenic mechanisms are not entirely understood; however, a hyperactive immune response, complement activation, and microvascular injury are hypothesized to contribute to cutaneous vasculitis [9]. Additionally, both histopathological features and clinical features have been specifically noted with many of the main cutaneous vasculitides associated with a prior COVID-19 infection. Common cutaneous vasculitides include cutaneous small-vessel vasculitis, urticarial vasculitis, skin-limited IgA vasculitis, and lymphocytic vasculitis [10]. In a case series, Corrá et al. reported that the timeline from having COVID-19 to developing vasculitis varies, ranging from within a few days to over 30 days [11]. In terms of cutaneous vasculitis post-COVID-19 vaccine, onset varies based on the vaccine dose and length after the dose. In the same case series, it was reported that patients developed cutaneous vasculitis four hours after the first dose to 17 days after the first dose, while other individuals developed blanchable purpura one day to four weeks after the second dose [11]. In the elderly population (age > 65) specifically, the case series reported 10 different cases of individuals from 65 years old to 91 years old. The onset of cutaneous vasculitis occurred seven to 10 days after the first dose, five to 12 days after the second dose, and two days to two weeks after the third dose [11]. While recent studies have discovered associations between prior COVID-19 infection and cutaneous vasculitis and vasculopathy, further research is needed to determine the pathophysiology of these findings.

With COVID-19 causing various types of vasculitis, an increase has been noted in giant cell arteritis (GCA) accompanying post-COVID syndrome. Giant cell arteritis is a common primary systemic vasculitis that may lead to irreversible vision loss, and a disruption of the healthcare system caused by the COVID-19 pandemic uncovered weak points clinically for the diagnosis and treatment of giant cell arteritis [12]. Many overlapping features of GCA and COVID-19 infection are seen in the beginning, including headache, fever, elevated C-reactive protein (CRP), and cough, while jaw claudication, visual loss, platelet count, and lymphocyte count may be more discriminatory between COVID-19 infection and GCA [13]. If patients are seen having a combination of these symptoms while in the infectious stage of COVID-19 or in patients with post-COVID syndrome, it is imperative to work these patients up for GCA to rule out a secondary inflammatory response to the temporal artery.

In regard to the temporal artery, research has been conducted on how the delayed diagnosis of COVID-19 has contributed to an increased rate of preventable bilateral blindness in giant cell arteritis in Italy since the outbreak of the COVID-19 pandemic, as compared to 2019 [14]. It was discovered that patients who had been previously diagnosed with COVID-19 were more likely to experience symptoms that were indicative of GCA, such as headache, vision changes, increased CRP level, and findings from a temporal artery biopsy or high-resolution MRI. Specifically, a substantial increase in the incidence of patients with GCA during the COVID-19 pandemic was seen as compared with those diagnosed during the same time period in 2019 [14]. In this particular study, 17 cases of GCA were diagnosed since the onset of the COVID-19 pandemic, as compared with 10 in 2019, a 70% increase without any demographical, clinical, or biological differences between the groups [14].

Another study found similar results regarding an increase in cases of GCA after the COVID-19 pandemic began, namely due to a lack of follow-up care or reluctance to seek medical care out of fear of seeking treatment in a public environment. Specifically, after the outbreak of SARS-CoV-2, there has been a decline in requests for fast-track assessments by 75% compared with the same time in 2019 [15]. It has been suggested by multiple institutions that more research is needed in order to compare the similarities between COVID-19 secondary vasculitis and true GCA and to provide an efficient and effective treatment for possible GCA caused as a secondary manifestation of COVID-19. Early diagnosis of post-COVID syndrome vasculitis is critical in order to prevent morbidity and complications of GCA, in addition to preventing blindness.

Furthermore, a case report found a man with a history of COVID-19 infection prior to presenting with a scotoma. This case presented a 47-year-old man with a history of COVID-19 infection two months before presentation who presented with a scotoma of the paracentral visual field of the right eye [16]. This man was found to have a diagnosis of paracentral acute middle maculopathy before subsequently developing a temporal headache as well as jaw claudication. The patient was worked up for GCA, given high-dose steroids, and experienced a resolution of symptoms within 24 hours post-steroid initiation [16]. This may have been the first patient to potentially implicate COVID-19 in ophthalmic vasculitis.

A separate case study found a man with mild COVID-19 symptoms who presented to his dermatologist with fever, cough, temporal artery thickening, and a headache. Due to his recent COVID-19 infection, he was not worked up for giant cell arteritis because of the overlap of GCA symptoms with COVID-19 symptoms. After continuing to experience persistent headaches and jaw pain, he was worked up further for an alternative diagnosis. On the exam, his blood test was positive for both COVID-19 immunoglobulin G (IgG) and IgM. Erythrocyte sedimentation rate (ESR) and CRP were normal; however, ultrasound of the right temporal artery implied arterial wall thickening and inflammation based on the appearance of the halo sign [17]. Additionally, a positron emission tomography (PET) scan revealed an increase in metabolic activity in the abdominal aorta. This patient improved spontaneously with the resolution of the arterial wall inflammation and improved blood flow [17]. The most likely diagnosis supporting this evidence was GCA triggered by the SARS-CoV-2 infection. While there is a lack of confirmation of this diagnosis based on the novelty of the disease correlations, COVID-19 and GCA definitively have overlapping symptoms that have repeatedly been reported. More research is needed to determine if SARS-CoV-2 is a virus-precipitating disease of GCA.

Evidence from a research center in the United Kingdom has shown an increase in the number of patients diagnosed with GCA and an increase in the number of visual complications following the COVID-19 pandemic. These researchers found that in the 12-week period between April and June 2020, there were 24 patients diagnosed with GCA. Six (25%) had visual impairments. In contrast, in 2019, 28 new diagnoses of GCA were made, and 10% of patients had visual impairment [18]. The number of patients from this study during April-June 2020 was fivefold higher than the prior year before the onset of the COVID-19 pandemic. Of note, a greater proportion of male patients and a decreased median age were seen, but there was no obvious difference in the duration of symptoms before the assessment [18]. These findings, amongst other similar studies worldwide, suggest that a viral pathogenetic hypothesis could exist for GCA. Research in immunology and rheumatology has been considered urgent in providing patients with the proper diagnosis and treatment during their active, acute COVID-19 infection or throughout a more chronic state of post-COVID syndrome.

Genetic studies have demonstrated that human leukocyte antigens (HLAs) DRB1*04:04 and B*15:01 increase the risk of cranial and extracranial GCA [19, 20]. In a case series conducted by Stojanovic et al., researchers tested for the HLA DRB1*04 in patients who developed a large vessel GCA after having COVID-19 [21]. In all three case studies, the patients did not have the DRB1*04 allele; however, the presence of HLA-DRB1*15 was identified in two of the patients [21]. This is in contrast to previous findings that HLA-DRB1*15 carries a protective effect [20, 21]. Further studies are needed to determine the role of the HLA allele in the pathology of GA.

Another large vessel arteritis, Takayasu arteritis (TA), has also been linked to post-COVID syndrome [22]. One case report documented a 13-year-old female who presented to the emergency department with chest pain and syncope [22]. While the patient was never tested for COVID-19, her uncle, whom she lived with, had COVID-19 about six months ago [22]. Vitals included blood pressure discrepancies: 128/71 mmHg in the right arm, 122/72 mmHg in the left arm, versus 106/67 mmHg in the right leg, and 107/74 mmHg in the left leg [22]. Labs revealed positive anti-double-stranded deoxyribonucleic acid antibodies (anti-dsDNA) and negative antineutrophil cytoplasmic antibodies (anti-cANCA) with elevated acute phase reactants [22]. In terms of imaging, the patient had pericardial and pleural effusion on a CT scan, as well as mural thickening in the descending thoracic aorta to the suprarenal level [22]. The patient was started on IV methylprednisolone at 30 mg/kg/day for three days and discharged on oral prednisolone, methotrexate, and anti-tumor necrosis factor (TNF) therapy [22]. In a similar case report, a 17-year-old female presented with two months of back pain, a dry cough, weight loss, and a fever of two days [22]. The patient also had blood pressure discrepancies: 94/59 mmHg in the left arm, 103/60 mmHg in the right arm, 125/77 mmHg in the left leg, and 132/80 mmHg in the right leg [22]. On a physical exam, the patient also had a 1/6 systolic murmur in the aortic focus [22]. There was an increased F-fluorodeoxyglucose (FDG) uptake in the common carotids, aortic arch, and ascending and descending aortic walls on PET-CT [22]. An MR angiography revealed enlargement and stenosis of the aortic arch, a wide ascending aorta, stenosis in the left subclavian artery, and the origin level of the left renal artery [22]. The patient was diagnosed with TA and treated with IV methylprednisolone at 30 mg/kg/day for three days [22].

Additionally, post-COVID syndrome has implications for causing inflammation leading to aortitis. In addition to the numerous cases of COVID-19 causing small-medium-sized vasculitis, large vessel vasculitis, specifically aortitis, has now been reported in COVID-19-positive patients. The first report of aortitis occurred in a 71-year-old COVID-19-positive man who presented with dry cough, fever, diarrhea, and dyspnea in March 2020 [23]. These initial symptoms resolved within two weeks, but he later presented with fatigue, weight loss, and left-sided chest pain radiating to the scapulae [23]. The CT demonstrated diffuse, inflammatory aortitis from the subclavian arteries to the iliac bifurcation without dissection or pseudoaneurysm formation [23]. Forty milligrams of prednisolone were prescribed, which alleviated symptoms within two weeks and resolved this inflammatory process [23].

In a second case study of a 63-year-old COVID-19-positive man, he initially presented with diffuse abdominal pain, dry cough, fatigue, and weakness for two days without shortness of breath, hypoxia, body rash, jaw claudication, or joint pain [24]. Labs recorded an elevation of ESR at 57 mm/hr, CRP at 8.7 mg/dl, and IL-6 at 54.44 pg/mL with negative infectious vasculitis serology [24]. A CT of the abdomen and pelvis showed inflammatory changes in the infra-renal aorta with a small aortic dissection. After determining the aortitis was secondary to COVID-19, the patient was started on 60 mg of prednisone, which resolved all aortitis symptoms.

A third case study demonstrating an association between post-COVID syndrome and aortitis included a 19-year-old female who presented with fatigue, malaise, and chest and back pain [25]. She endorsed a previous COVID-19 infection one month prior to her presentation. Laboratory tests showed an elevated ESR (109 mm/hr), increased CRP (10.73 mg/dL), anemia (hemoglobin 10.4 g/dL), and a negative autoimmune work-up. A CT of the chest, abdomen, and pelvis revealed concentric and diffuse thickening of the descending thoracic and abdominal aorta, with signs of thickening of the common iliac and renal arteries [25]. This inflammatory activity was confirmed by FDG-PET [25]. The patient was started on prednisolone 60 mg/day, and their symptoms improved.

The proposed mechanism of COVID-19-induced aortitis is through SARS-CoV-2 accessing the endothelial cells through angiotensin-converting enzyme-2 receptors driving T helper cells, activating cell-mediated immunity and humoral immunity, which leads to endotheliitis and leukocytoclastic vasculitis, as well as type-3 hypersensitivity reactions causing aortitis [24]. This inflammation can increase ESR, CRP, and IL-6, making those biomarkers good indicators of disease progression. Both cases responded well to prednisone after ruling out any other infectious or noninfectious causes of aortitis. Although these cases were rare, it is important to be clinically suspicious of aortitis when COVID-19-positive patients present with nonspecific abdominal or chest pain. This life-threatening diagnosis can occur at the onset of symptoms or later in the clinical course, making this condition even more difficult to diagnose.

An additional type of vasculitis seen in patients with inflammation from a previous COVID-19 infection is Kawasaki-like disease. In children, multiple cases of Kawasaki-like disease have been identified in COVID-19-positive patients. Kawasaki disease is a small- and medium-sized vasculitis that predominantly affects children, with rare cases seen in adults. The cause of Kawasaki and its relationship to COVID-19 is still unclear, but the proposed mechanism involves SARS-CoV-2 binding to ACE2 STING pathway activation, and during the second phase of illness with immune hyper-responses, there have been decreased lymphocyte counts and increased monocyte populations that secrete cytotoxic cytokines and heightened B and T cell responses [2]. These patients presented with persistent high fevers, chilblain-like lesions, and rashes, with severe forms causing complications in coronary artery aneurysms, the rupture of which leads to thrombosis formation, myocardial infarction, and cerebral artery aneurysms [26]. These complications can be avoided with early diagnosis and treatment. One case study of a six-month-old baby diagnosed with Kawasaki disease and COVID-19 received intravenous immunoglobulin (IVIG) and aspirin, leading to the alleviation of symptoms [26]. In another rare case of adult-onset Kawasaki disease in a COVID-19-positive patient, the patient was diagnosed early (< four days) and treated with IVIG and corticosteroids, reducing the development of coronary arterial aneurysms and improving left ventricular performance and capacity [2]. Due to the importance of early diagnosis and treatment, it is critical for patients with Kawasaki disease symptoms to seek medical care and be tested for COVID-19. These patients should also warrant a close follow-up to investigate the delayed presentation of coronary or cerebral aneurysms to potentially decrease the rate of late complications.

Medium- and large-vessel vasculitis have been documented more frequently as they relate to post-COVID syndrome and infection. Numerous cases of postinfectious vasculitis have been reported in the literature during and after the COVID-19 pandemic. Furthermore, vasculitis changes have been seen in radiological studies in COVID-19 survivors with persistent symptoms. One study evaluated several patients with unexplained persistent symptoms after SARS-CoV-2 with 2-deoxy-2 [18F]fluoro-D-glucose (^18^FDG/FDG) in order to demonstrate a persistent inflammatory process [27]. This particular study found that the target-to-blood pool ratio was substantially higher in three specific vascular regions (femoral arteries, right iliac arteries, and thoracic aorta) in patients in the recovered COVID-19 cohort versus the controls. This suggests that SARS-CoV-2 contributes to vascular inflammation, which leads to persistent vascular symptomatology [27].

## Conclusions

Both active and chronic SARS-CoV-2 infections have the potential to lead to secondary inflammation that may cause varying types of small-, medium-, and large-vessel vasculitis. While limited research has been conducted on vasculitis secondary to COVID-19 infection, preliminary data and case studies suggest that both acute and chronic SARS-CoV-2 infection may lead to specific diseases such as giant cell arteritis, ophthalmic arteritis, aortitis, and Kawasaki-like disease. More research should be conducted on the immunological aspects of COVID-19 infection and blood vessel involvement in order to formulate a better understanding of the correlation between COVID-19 infection, post-COVID syndrome, and secondary vasculitis due to viral inflammation. This type of research would contribute greatly not only to disease pathogenesis but also to the accurate, timely diagnosis and treatment of many different types of vasculitis brought about by the SARS-CoV-2 virus.
